# Low dose of IL-2 combined with rapamycin restores and maintains the long-term balance of Th17/Treg cells in refractory SLE patients

**DOI:** 10.1186/s12865-019-0305-0

**Published:** 2019-09-04

**Authors:** Chunmiao Zhao, Yanfang Chu, Zhaoyun Liang, Bingying Zhang, Xuxia Wang, Xiaona Jing, Meihua Hao, Yiqi Wang, Jia An, Xingzhe Zhang, Liguang Sun, Junwei Chen

**Affiliations:** 1 Deptartment of Rheumatology and Immunology, The Second Hospital of Shanxi Medical University, Taiyuan, 030001 China; 2 School of Basic Medical Sciences, Xi’an Jiaotong University, Xi’an, 710061 China; 3 Institute of Translational Medicine, The First Hospital of Jilin University, Changchun, 130021 China

**Keywords:** Systemic lupus erythematosus, IL-2, Rapamycin, Th17 cells, Treg cells

## Abstract

**Background:**

The development of Systemic lupus erythematosus (SLE) has been associated with the balance of Th17 and Treg cells. IL-2 and rapamycin can influence the populations of both Th17 and Treg cells. However, it is unclear whether low dose of IL-2 and rapamycin can relieve the symptoms of SLE patients and what is the mechanisms. In this study, we aim to analyze the effect of low dose of IL-2 plus rapamycin on the number of Tregs, Th17 cells and the ratio of Th17/Treg cells, as well as to evaluate its therapeutic efficacy in refractory SLE patients.

**Result:**

Fifty refractory SLE patients and 70 healthy controls were enrolled and followed up for 24 weeks. We found that compared with HC, the refractory SLE patients had a lower number of Tregs, a similar number of Th17 cells, but an increased ratio of Th17/Treg. After the treatment, the number of Tregs of the patients at 12th and 24th week was significantly increased. While the number of Th17 cells was unchanged, the ratio of Th17/Treg was significantly decreased at both 6 weeks and 24 weeks. After 6, 12 and 24 weeks of treatment, the SLEDAI score was significantly reduced. The prednison dosage at 6th,12th and 24th week post treatment was significantly decreased.

**Conclusion:**

Our results support that the reduction of Tregs and the imbalance of Th17/Treg cells were correlated with the occurrence and development of refractory SLE. Low dose of IL-2 combined with rapamycin was able to restore the number of Tregs and the balance of Th17/Treg cells. As a result, this approach was able to induce immune tolerance and promote disease remission, allowing for the reduction in prednisone dosage.

**Trial registration:**

ChiCTR-IPR-16009451 Registration date: 2016/10/16

## Background

Systemic lupus erythematosus (SLE) is a type of autoimmune diseases involving multiple systems and organs injury. An increasing number of studies have shown that T cells play an important role in the developmental process of SLE [1]. T-helper 17 cells (Th17) can induce tissue inflammation and immune responses, while regulatory T cells (Tregs) can mediate immune tolerance as well as inhibit tissue inflammation and immune response. The imbalance of Th17 cells and Treg cells influences the initiation and pathogenesis of SLE [2]. An increased number of Th17 cells and a decreased number of Treg cells are correlated with the occurrence of SLE [3]. Therefore, inhibiting Th17 cells differentiation and promoting Treg cells differentiation can restore the balance of Th17/Treg cells and become a potential approach to treat SLE. Recent studies found that low dose of IL-2 can promote the number of Treg in T1D [4], HCV-induced vasculitis [5], GVHD [6, 7] and alopecia areata [8]. In addition, low dose of IL-2 can increase the number of Treg cells and maintain immune homeostasis transiently to alleviate disease symptoms in SLE patients [9–13], however, majority of the studies are focused on the short-term effect [13] which is not a feasible long term solution [14]. While rapamycin can inhibit the differentiation of Th17 cells and promote the proliferation and the differentiation of Treg cells, such as in vitro and in vivo in kidney transplant recipients [15], a murine CD4^+^ T cell transfer model of colitis and an experimental autoimmune encephalomyelitis (EAE) model [16] and CCl4-induced liver fibrosis [17]. Therefore, it is unknown whether low dose of IL-2 combined with rapamycin can increase the number of Tregs and restore the balance of Th17/Treg cells as well as maintain the function long term in SLE patients, although we did a limited primary observation [18]. In this study, we will evaluate the effect of low dose IL-2 and rapamycin on the Treg status and immune balance.

## Methods

### Patients and healthy controls

Total 50 patients with refractory SLE, including 47 females and 3 males, were enrolled in the department of Rheumatology, the 2nd Hospital of Shanxi Medical University, from December 2016 to January 2018. A detailed list of characteristics of the patients were shown in Table 1. All the patients were diagnosed according to the classification standard revised by the American College of rheumatology (ACR) in 1997 [19]. Refractory SLE refers to the disease continues, but does not alleviate or worsen in clinical and laboratory indexes after the induction with high-dose glucocorticoid (> 1 mg. Kg-1. D-1) and/or more than 1 cytotoxic drug [20, 21]. We eliminated patients with the complication of serious heart, liver, kidney disease, vital sign instability and contraindications and allergic histories of IL-2 and rapamycin from the study. Patients who are participating in other clinical trials were also eliminated; patients with tumors, infections, pregnancy or lactation, renal insufficiency were further eliminated from the study. Clinical and laboratory data were collected in detail for all patients, and the activity score (SLEDAI) was determined according to literature [22]. Seventy age-matched (35.84 ± 9.40 years) and gender-matched healthy adults were selected as healthy controls, including 59 females and 11 males. All subjects agreed to participate in this study voluntarily and signed the consent form.

**Table 1:** Demographic and clinical characteristics of systemic lupus erythematosus patients

Characteristic	Value
Age,years,mean±SD	33.10±11.72
Gender,female,n(%)	47(94%)
Male,n,(%)	3(6%)
Duration,months,mean±SD	74.22±40.91
ESR(mm/h),mean±SD	21.24±18.81
SLEDAI,mean±SD	5.94±2.63

### Method

Low dose of IL-2 (100 WIU, 3-5d/month, subcutaneous injection) and rapamycin (0.5 mg, once every other day, oral) were given to all refractory SLE patients. The following data were collected and analyzed: clinical characteristics, medication status, ESR, and the absolute number and ratio of Th17 and Treg cells. Data for refractory SLE group and healthy control group were compared, and the therapeutic efficacy of low dose of IL-2 plus rapamycin in the treatment of refractory SLE was analyzed.

### Flow cytometry analysis

Peripheral blood mononuclear cells (PBMCs) were isolated from heparinized peripheral blood by Ficoll-Paque PREMIUM 1.077 (GE Healthcare Life Sciences, Pittsburgh, PA) density gradient centrifugation. 2X10^6^ cells were cultured in RPMI 1640 medium with 50 ng/ml PMA, 1 μg/ml ionomycin and 1 μl Golgi Stop for 5 h in a CO_2_ incubator at 37 degree. The stimulated cells were stained with FITC-anti-human CD4 Ab for 30 min at 4 degree. After washing, the cells were incubated with freshly prepared Fixation/Permeabilization solution at 4 degree for 30 min in the dark. After washing, the fixed cells were stained with PE-anti-human IL-17 Ab (560436, BD Pharmingen™) at 4 degree for for 30 min in the dark. Then the cells were washed and analyzed by flow cytometry.

### Intracellular staining

PBMCs were incubated with FITC-anti-hCD4(555346,BD Pharmingen™) and APC-R700 anti-hCD25 Abs(565106,BD Horizon™) at 4 degree for 30 min in the dark. Then cells were treated with 1 ml freshly prepared Fixation/Permeabilization solution at 4 degree for 30 min in the dark, and then stained with PE-anti-human FOXP3 antibody(560046,BD Pharmingen™) at 4 degree for 30 min in the dark, washed with PBS, and detected by a flow cytometer (Calibur, BD, USA). Cells were analyzed with a BD FACSCanto II system (BD Biosciences, San Jose, CA), followed by data analysis using FlowJo 7.6 software (TreeStar, SanCarlos, CA).

### Statistical analysis

The data were analyzed by SPSS 20.0 statistical software (SPSS Inc., Chicago, IL) and GraphPad Prism 5.0 software (GraphPad Software 5.0, San Diego, CA). Kolmpgorov-smirnov test and Levene’s T test were used for normal test and variance homogeneity analysis. Two-group comparison was performed with student’s t test or the nonparametric Mann-Whitney rank-based test. For > 2 groups, one-way analysis of variance (ANOVA) or the nonparametric Kruskal-Wallis test was performed, and the pairwise comparison was conducted using the LSD test. Pearson test was used for correlation analysis. *P* < 0.05 was considered as significantly different.

## Results

### Decreased Tregs number in the PBMCs of SLE patients

**Fig. 1:**
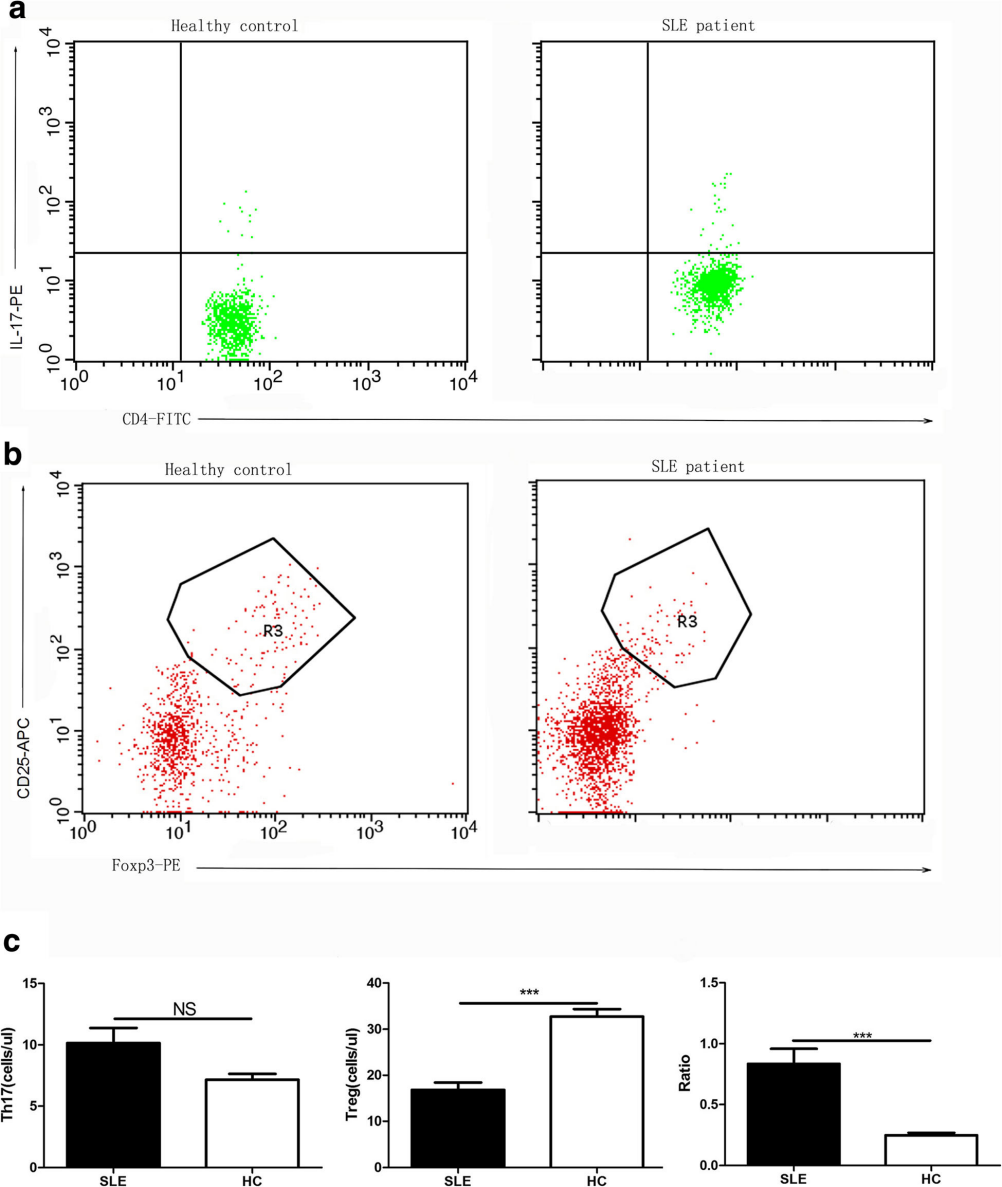
FACS analysis of Th17 cells and Treg cells in the PBMCs of refractory SLE patients and healthy controls. **a** Representative flow cytometric analysis of human Th17 cells (CD4 ^+^ IL-17^+^) isolated from healthy control and SLE patients; **b** Representative flow cytometric analysis of human Treg cells (CD4 ^+^ CD25 ^+^ Foxp3+) isolated from healthy control and SLEpatients; **c** The absolute number of Th17 cells and Treg cells; The ratio of Th17 cells /Treg cells. N_SLE_ = 50; N_HC_ = 70

To assess the changes of Tregs and Th17 cells in SLE patients, we analyzed the total cell numbers of these two populations by FACS. We found that while the absolute number of Th17 cells was not significantly different (*P* = 0.157) between the two groups, the absolute number of Treg cells in refractory SLE patients was significantly lower than that of the healthy controls (*P* < 0.001). In addition, the ratio of Th17/Treg cells was significantly higher than that of the healthy control group (*P* < 0.001) (Fig. 1a, b and c).

### No correlation between the absolute number of Th17 cells, Treg cells, Th17/Treg ratio, the duration of disease, SLEDAI score and ESR in refractory SLE patients

Through pearson test analysis, we did not observe significant correlation of the absolute number of Treg cells, Th17 cells, Th17/Treg ratio with disease duration, SLEDAI score and ESR (Table 2).

**Table 2:** Associations of Treg, Th17 cells and Th17/Treg with disease duration, SLEDAI and ESR

group	duration		ESR		SLEDAI	
	r	p	r	p	r	p
Th17	0.110	0.451	0.152	0.297	-0.010	0.944
Treg	-0.231	0.110	-0.269	0.062	-0.267	0.063
Th17/Treg	0.267	0.064	0.236	0.103	0.001	0.992

### Changes in disease activity indicators, Th17, Treg, Th17/Treg, prednison and DMARDs in patients with refractory SLE during the follow-up period

#### Low-dose IL-2 combined with rapamycin increased Treg cells population in refractory SLE patients

**Fig. 2:**
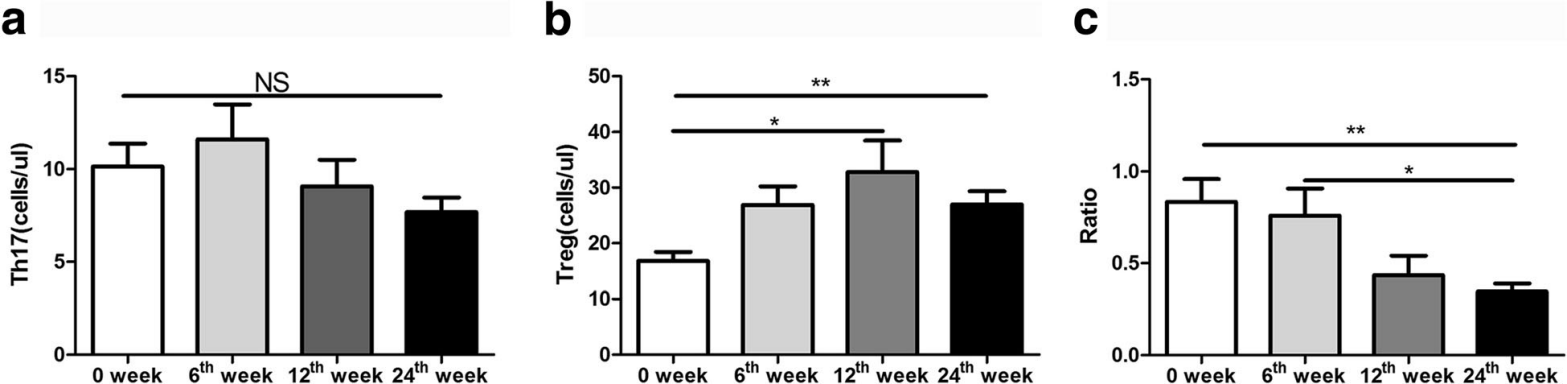
Effect of low-dose IL-2 combined with rapamycin on the populations of Th17 and Treg cells: **a** The number of Th17 cells; **b** The absolute number of Treg cells; **c** The ratio of Th17/Treg One-way analysis of variance (ANOVA) test was performed, and the pairwise comparison was conducted using the LSD test. N_SLE_ = 50

Since it is known that low-dose IL-2 and rapamycin can increase the proportion of Treg cells, we assessed the effect of the combined treatment in patients with refractory SLE. We found that during the follow-up period after treatment, although the absolute number of Th17 cells was unchanged (*P* = 0.107), the absolute number of Treg cells significantly increased at 12 and 24 weeks (*P* = 0.015 and *P* = 0.002). Moreover, the ratio of Th17/Treg significantly decreased at 24 weeks post-treatment (*P* = 0.007) (Fig. 2a, b and c).

#### SLEDAI significantly decreased after the treatment of low-dose IL-2 combined with rapamycin

**Fig. 3:**
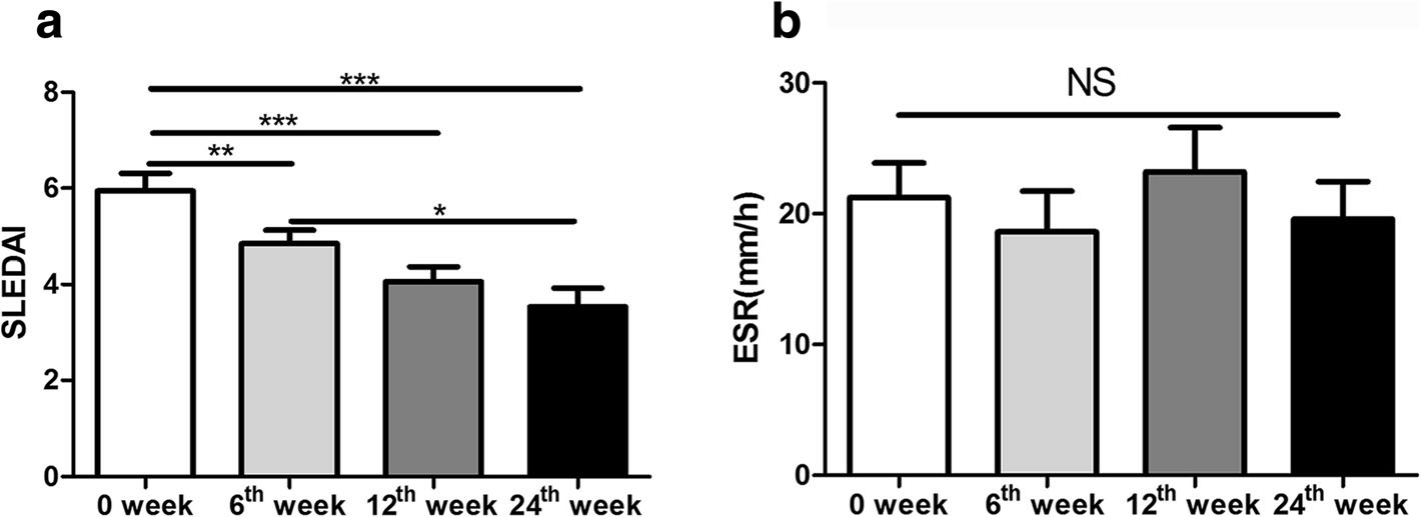
Effect of low-dose IL-2 combined with rapamycin on disease parameters in patients with refractory SLE: **a** The SLEDAI score analysis; **b** The ESR analysis One-way analysis of variance (ANOVA) test was performed, and the pairwise comparison was conducted using the LSD test. N_SLE_ = 50

After 24 weeks of treatment, the SLEDAI score was significantly lower than 0 week(*P*<0.001) and 6 weeks (*p* = 0.012). The SLEDAI score at 6th week was lower than 0 week(*P* = 0.002). The SLEDAI score at 12th week was significantly lower than 0 week(*P*<0.001). There was no statistical significance in ESR observed during the follow-up period (*P* = 0.304) (Fig. 3a and b).

#### Prednison dosage significantly decreased after the treatment of low-dose IL-2 combined with rapamycin

**Fig. 4:**
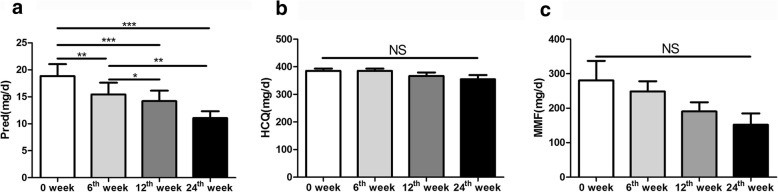
Effect of low-dose IL-2 combined with rapamycin on drug dose in patients with refractory SLE. **a** The change of prednison dosage at 0, 6th,12th and 24th week. Prednison; **b** the HCQ change at different week post treatment; **c** the MMF change at different week post treatment. One-way analysis of variance (ANOVA) test was performed, and the pairwise comparison was conducted using the LSD test. N_SLE_ = 50

During the 24-week follow-up period, prednison dosage at 6th,12th and 24th week was significantly lower than that of 0 week(*p* = 0.008, *p*<0.001 and *p*<0.001). The prednison dosage at 12th and 24th week was significantly lower than that of 6th week(*p* = 0.022 and *p* = 0.005). By contrast, the DMARDs drug dosage did not decreased(*p*_HCQ_ = 0.228 and *p*_MMF_ = 0.290) (Fig. 4a, b and c).

## Discussion

The developmental process and mechanism of SLE are highly complicated. Initial studies have shown that SLE is associated with immune disorders caused by the overactive T-cell-dependent B cells. Futhermore, it has been demonstrated that Th17 cells and Treg cells are involved in the pathogenesis of SLE [2, 3].

Th17 cells are a subgroup of CD4^+^T cells producing cytokine IL-17. In the presence of TGF-β and IL- 6, Naive T cells express retinoic acid-related orphan nuclear receptor-γt (RORγt) [3] and differentiate into Th17 cells. The differentiation of Th17 cells is regulated by an activated mTOR, a conserved serine/threonine kinase. Tregs are also known as CD4^+^CD25^+^Foxp3^+^ T cells, which are primarily differentiated from native T cells in the presence of TGF-β [23]. Regulated by the STAT5 signaling pathway, Tregs play an important role in maintaining immune balance and regulating immune tolerance. A number of studies have shown that Th17 cells are significantly increased in both SLE mice and patients [24]. In the target organs of SLE patients, the number of Th17 cells as well as the expression level of IL-17 in peripheral blood are increased, which is correlated with disease activity [25]. This suggests that Th17 cells and IL-17 production are associated with the progression of SLE. Recently, many clinical studies on human autoimmune diseases, animal models and SLE patients revealed the decrease of Treg cells number is closely related to the disease occurrence [3, 26]. In this study, we also found that in comparison with the healthy control group, refractory SLE patients have a reduced Treg number in peripheral blood, which is consistent with the results from Noack’s study [26], suggesting that the pathogenesis of SLE may be related to the destruction of autoimmune tolerance caused by a decrease of Treg. Yang and colleagues found that the underlying pathogenesis of lupus mice might be induced by the imbalanced ratio of Th17/Tregs and cytokines in the microenvironment [27]. Consistently, Doff and colleagues demonstrated that the ratio of Th17/regulatory T cells in patients with the remission of SLE was higher than that in healthy controls [28]. In this study, we also found the ratio of Th17/Treg in refractory SLE group tended to be lower compared with that in healthy controls although there was no statistical significance. Furthermore, the lack of difference in Th17 cells between refractory SLE group and healthy control group, suggests that the reduction of Treg cells may be the primary reason for the imbalance of Th17/Treg cells in SLE patients, leading to the breakdown of autoimmune tolerance, contributing to the occurrence and development of SLE.

Since we have demonstrated that Th17/Treg imbalance is associated with the occurrence and development of SLE, we speculated that this may be associated with the disease index. In this study, we collected all the patients’ detailed characteristics, clinical and laboratory data to analyze their association with Th17/Treg imablance. However, all results demonstrated that there is no significant correlation across the absolute number of Treg cells, Th17 cells, Th17/Treg ratio with disease duration, SLEDAI score and ESR. However, Qian Xing et al. found that Th17/Treg is related to SLEDAI scores [25]. The reason for this difference may be that we pay more attention to the remission of refractory SLE patients but the difference in disease activity has been overlooked. It has been reported that Th17 cells in the active stage of the disease were significantly higher than those in the control group, while Th17 cells in the non-active stage were not significantly different from those in the control group [27]. In our study, there were patients who maintained a low level of activity without continuous remission, leading to a lack of statistical difference in the results.

For the role of Th17/Treg imabalance in the occurrence and development of SLE, It is crucial to restore and maintain the balance. It is well known that the differentiation and amplification of Treg cells require the presence of IL-2, and IL-2 can induce Foxp3 expression [29, 30]. The level of IL-2 affects the immune tolerance mediated by Treg cells, while low dose of IL-2 can selectively activate Treg in T1D [4], HCV-induced vasculitis [5], GVHD [6, 7], alopecia areata [8] and SLE [31]. IL-2 can also inhibit the differentiation of Th17 cells by inhibiting the expression of RORγt through the activation of STAT5 [32]. Therefore, the treatment of low-dose IL-2 for SLE can restore the balance of Th17/regulatory T cells and achieve immune tolerance, which may alleviate the condition of SLE patients. Von Spee-Mayer C et al. treated five refractory SLE with low-dose IL-2 for 5 days and found that Treg were increased in all patients. However, the study did not discuss the long-term effects [13]. Humrich et al. applied IL-2 to treat a patient with refractory SLE patient resulting in the rapid alleviation of the patient’s condition. However, at 6th week, the patient condition worsened: the SLEDAI score increased to 10 while the number of Treg cells decreased [14]. The above findings suggest that IL-2 can increase the number of Treg cells to improve condition promptly in SLE patients, but this effect was tempory. This could be explained by the notion that IL-2 can also interact with surface receptors on different cells to activate a variety of signaling pathways. Also, the biological effects mediated by IL-2 can be influenced by the concentration, the environment and the surrounding inflammatory cells [29, 33, 34].

Rapamycin is originally recognized as an antifungal drug, Kurebayashi et al. [16] confirmed that rapamycin can form an immunosuppressive compound with FKBP-12 (immunosuppressive protein) in cells, which can bind mTORC1 and block the signal transduction of PI3K/AKT/mTOR pathways. This can reduce the expression of RORγt, inhibiting the differentiation of Th17 cells while enhancing the transcription activity of Foxp3, Treg differentiation and function [35]. Also, in vitro studies revealed that rapamycin blocked Stat3 activation and greatly reduced the number of IL-17 effector cells [36]. Rapamycin can also induce the production of iTreg cells [37] by inhibiting mTORC1 to enhance the Smad3 signaling pathway, promoting the proliferation and differentiation of Treg cells [38]. Thus, in some studies, Rapamycin was used to inhibit the differentiation of Th17 cells and promote the proliferation and differentiation of Treg cells in kidney transplant recipients [15], murine CD4^+^ T cell transfer model of colitis and an experimental autoimmune encephalomyelitis (EAE) model [16] and CCl4-induced liver fibrosis [17]. If IL-2 is used along with rapamycin to treat SLE, it is possible that this approach not only promotes an increase in the Treg populationin SLE patients, but also overcomes the short-term effect. Th17 cells and Treg cells can reach a stable immune balance state, which can control the disease progression of SLE.

Based on the above studies, we adopted low-dose IL-2 in combination with rapamycin as a treatment for refractory SLE patients to analyze the numbers of Tregs and Th17 cells. Also, we observed the clinical indicators as well as the basic clinical medication to evaluate the drug efficacy in patients with refractory SLE. As for the dose of IL-2 and rapamycin, different studied utilized varying doses. However, most doses of IL-2 ranged from 1X10^5^-3X10^6^ IU/m^2^/day while the dose of rapamycin is 2 mg/day without the report of adverse effects [8, 12, 15, 10]. In this study, we administered IL-2 with 100 WIU, 3-5d/month via subcutaneous injection to SLE patients and rapamycin with 0.5 mg, once every other day by oral administration. No serious adverse events were found. In addition, the the number of Treg cells in patients treated with low-dose IL-2 combined with rapamycin at 12th and 24th week was significantly increased compared to 0 week. Humrich et al. only used IL-2 to observe that the number of Treg cells increased before 6 weeks, but decreased after 6 weeks. However, in our study, low-dose IL-2 combine with rapamycin could lead to an increase of Treg cells till the 24th week. Furthermore, the ratio of Th17/Treg was significantly lower at 24th week compared to that at 6th week, despite that there was no significant difference in the number of Th17 cells. Altogether, we conclude that the change in the Th17/Treg ratio is mainly caused by the change in the Treg population.

As for the clinical indicators, we observed that the SLEDAI gradually decreased after treatment. The SLEDAI score at 6th week and 12th week was lower than 0 week. After 6th week post treatment, the SLEDAI changed smoothly, which might account for the role of IL-2 but the maintenance mainly account for the use of rapamycin. Also, the SLEDAI at 24th week is 3.54 ± 2.73 suggesting that the disease is at the inactive stage. The SLEDAI scores at 0, 6th and 12th week are 5.95 ± 2.63, 4.84 ± 1.98 and 4.06 ± 2.17, which suggests that the disease is at the active stage. However, we didn’t find any difference in ESR at 0, 6th,12th and 24th week, which indicates that ESR could be influenced by other factors.

It is well recognized that in severe cases of refractory SLE, large dose prednison or cytotoxic drugs are used to treat patients. However, current treatments also cause many side effects such as obesity, osteoporosis, gastric ulcer and infection. A combination of different drugs may not only alleviate disease but also decrease the dose of prednison or cytotoxic drugs, which will be beneficial to the patients. In this study, we found that a combination of low-dose IL-2 and rapamycin could reduce the dosage of prednison, which is beneficial due to the reduction of side effects. Although there was no difference in hydroxychloroquine (HCQ) and Mycophenloate Mofetil Capsuels (MMF), we observed a declining trend in the dose of MMF. In the future, we will increase the sample size to reevaluate this finding.

The above findings suggest that low-dose IL-2 combined with rapamycin for the treatment of refractory SLE patients can increase the number of Treg, restore Th17/Treg balance, reduce disease activity and reduce the dose of prednison, which is beneficial for patients with refractory SLE.

## Conclusion

In summary, our study has confirmed that the imbalance between Th17 cells and Treg cells caused by the significant reduction of Treg cells may be one of the major causes of SLE. Modulating the balance between Th17 cells and Tregs can be used as a potential approach for the treatment of SLE. We found that low dose of IL-2 combined with rapamycin significantly increased the number of Treg cells and restored the balance of Th17/Treg. This led to the alleviation of SLE symptoms, improvements in the rate of disease remission, and reduction in the dose of prednison. Although our study highlighted a new strategy to SLE treatment, future studies are needed to elucidate the mechanisms for how IL-2 and rapamycin synergistically enhance the Treg population long-term.

## Data Availability

The majority of the data generated or analyzed during this study are included in this published article and its supplementary information files. Any other datasets used and/or analyzed during the current study are available from the corresponding author on reasonable request.

## Abbreviations

ACR: American College of rheumatology
HC: Healthy controls
IL-2: Interleukin-2
PBMCs: Peripheral blood mononuclear cells
SLE: Systemic lupus erythematosus
SLEDAI: SLE disease activity index
Th17: T-helper 17 cells
Treg: Regulatory T cells
